# Efficacy and Safety of the Melanocortin Pan-Agonist PL9643 in a Phase 2 Study of Patients with Dry Eye Disease

**DOI:** 10.1089/jop.2023.0056

**Published:** 2023-11-02

**Authors:** David Evans, Kenneth Kenyon, George Ousler, Michael Watson, Patrick Vollmer, Eugene B. McLaurin, Gail Torkildsen, Jason Winters, John Dodd, Robert Jordan, Stephen T. Wills, Carl Spana

**Affiliations:** ^1^ Total Eye Care, Memphis, Tennessee, USA.; ^2^ Tufts University School of Medicine and New England Eye Center, Boston, Massachusetts, USA.; ^3^ Ora, Inc., Andover, Massachusetts, USA.; ^4^ Vita Eye Clinic, Shelby, North Carolina, USA.; ^5^ Andover Eye Associates, Andover, Massachusetts, USA.; ^6^ Palatin Technologies, Inc., Cranbury, New Jersey, USA.

**Keywords:** conjunctival staining, dry eye disease, efficacy, melanocortin receptor agonist, PL9643, tear film breakup time

## Abstract

**Purpose:**

The melanocortin receptor pan-agonist PL9643, a potential therapy for ocular diseases, was investigated in a phase 2, 12-week study in patients with dry eye disease (DED).

**Methods:**

This was a placebo-controlled study evaluating efficacy and safety of thrice-daily PL9643. Placebo (vehicle) was similar to tears. Primary endpoints were intra-patient changes in inferior corneal fluorescein staining and ocular discomfort after 12 weeks. Secondary endpoints were changes in additional DED signs or symptoms. Multiple secondary endpoints were not adjusted for multiplicity. Patients with moderate or severe DED were analyzed in addition to the overall intent-to-treat (ITT) population.

**Results:**

In the ITT population (*n* = 160) the PL9643 group did not demonstrate significant treatment difference versus placebo at week 12/day 85 for the primary endpoints (*P* > 0.05). In patients with moderate or severe DED (*n* = 53), PL9643 treatment demonstrated either nominally significant (*P* < 0.05) or trending (*P* < 0.1) improvement over placebo in mean change from baseline at week 12/day 85 in several sign endpoints, including fluorescein staining in inferior, superior, corneal sum, and total sum regions; Lissamine Green staining in temporal, nasal, conjunctival sum, and total sum regions; and tear film breakup time. Conjunctival redness also showed (nonsignificant) improvement at week 12/day 85. There were no drug-related adverse events (AEs) and no drug-related discontinuations.

**Conclusions:**

PL9643 showed no significant efficacy for the ITT population; however, efficacy results across several signs and symptoms in the subpopulation of moderate to severe DED patients, the low number of ocular AEs, and no tolerability issues suggest that PL9643 shows promise as a therapeutic for DED.

Clinical Trial Registration number: NCT04268069.

## Introduction

The melanocortins are a family of hormone agonists that include α-β-γ-melanocyte-stimulating hormones (MSH) and adrenocorticotropin hormone (ACTH).^1,2^ Binding of these agonists to melanocortin receptors (MCRs) produces their biological effects of resolving inflammation, promoting tissue healing processes, and maintaining immunological homeostasis, which have been demonstrated in many models of disease, both *in vivo* and *in vitro*.^1,3–5^ There are 5 MCRs (MC1R-MC5R), and these are distributed widely among cell types such as neutrophils, monocytes, macrophages, and fibroblasts and in the central nervous system and peripheral tissues.^1,3,6^ MC1R, MC3R, MC4R, and MC5R bind all the natural melanocortin hormones, although they differ in their affinities. MC2R binds only ACTH.^1^

The melanocortin system is important in regulating immune activity in the eye as α-MSH, by binding to MC1R, MC3R, and MC5R receptors and, through its effect on macrophages, antigen presenting cells, and antigen-specific T cells, suppresses inflammation and promotes inflammatory resolution.^7^ For example, treatments that increase α-MSH levels either systemically or locally induce the resolution of inflammation in experimental models of uveitis.^8–10^ The melanocortin pathway is also involved in other biological roles within the ocular system such as maintaining corneal transparency, sustaining the integrity and protection of the corneal epithelium, potentially improving donor corneal graft survival, regulating aqueous tear secretion, maintaining blood-retinal barriers, and controlling abnormal new vessel growth in the choroid and retina.^7^

The melanocortin pathway is therefore a potentially attractive target for new, nonsteroidal alternatives for the treatment of inflammatory eye diseases such as dry eye disease (DED) and uveitis, in addition to treating diabetic retinopathy and it has a role in aiding successful corneal transplantation.^7,11^ Synthetic melanocortin agonists, in particular those targeting MC1R and MC5R, have the potential to treat ocular pathologies with similar or better efficacy than glucocorticoids, but with a better safety profile since they may have greater potency, improved pharmacokinetics, and distinct receptor affinities.^4,7,11^

Dry eye is a multifactorial disease of the ocular surface characterized by a loss of homeostasis of the tear film, and accompanied by ocular symptoms, in which tear film instability and hyperosmolarity, ocular surface inflammation and damage, and neurosensory abnormalities play etiological roles.^12,13^ Many physicians and patients often regard existing dry eye therapies as inadequate owing to poor response, adverse side effects, poor ocular tolerability, and prolonged interval preceding therapeutic activity.^14–16^ PL9643 (Palatin Technologies, Inc., Cranbury, NJ) is a synthetic MCR pan-agonist (not active at MC2R) that is currently being investigated as a treatment for inflammatory ocular diseases. Here we describe the results of a phase 2 efficacy and tolerability clinical trial of PL9643 in adults with DED.

## Methods

### Ethics

The protocol, its amendments, and all relevant forms were reviewed and approved by a properly constituted independent ethics committee or institutional review board. The study was performed in accordance with the ethical principles of the Declaration of Helsinki and International Conference on Harmonisation Good Clinical Practice guidelines. All local, state, and federal requirements relevant to the use of investigational agents were followed. All patients provided written consent to participate, and informed consent was obtained before any study-related procedures.

### Study design

This was a phase 2, multicenter, randomized, placebo-controlled, double-masked study that evaluated the efficacy and safety of PL9643 in patients with DED. The study explored a range of signs and symptoms of DED using sign and symptom measures commonly used in clinical trials for DED in those patients who were classified as having mild, moderate, or severe DED.

The controlled adverse environment (CAE^®^) model was used to provide a consistent regulated environmental setting to exacerbate the signs and symptoms of DED. The CAE model is a controlled environment specifically developed to standardize the assessment of the signs and symptoms of DED. It consists of a chamber containing a standardized atmosphere in which subjects sit. This atmosphere has low humidity and increased airflow, and the subject undergoes various constant visual tasks. This environment consistently exacerbates dry eye signs and symptoms, which can then be measured. The dry eye measures are assessed immediately before and after the 90-min challenge. This model has been used for a number of clinical trials for both dry eye and other eye diseases.^17^ Assessments of signs and symptoms before, during, and after this challenge provided data on the extent of damage to the ocular surface, the extent and timing of recovery from that damage, and the effect of treatment on baseline response to adverse stimuli. During screening, two 90-min exposures to the CAE were conducted to ascertain eligibility to enter the study.

Patients were randomized 1:1 to 1 μg/mL PL9643 or placebo ophthalmic solutions to be taken bilaterally 3 times daily for 12 weeks. The placebo vehicle contained all the same excipients (trisodium citrate, dehydrate [2.79 mg/mL]; anhydrous citric acid [0.10 mg/mL]; sodium chloride [9 mg/mL]; polysorbate 80 [Tween, 1 mg/mL]; pH adjusted to pH 6.5) used in the active formulation without the PL9643 peptide. Anything less optimal than this vehicle (which has a similar pH and osmolarity to ophthalmic solution/natural tears) would cause tolerability issues with the vehicle-treated population which would make the study drug look artificially good in comparison.

The dose of 1 μg/mL was derived from preclinical studies with the MCR pan agonist PL8331 (Palatin Technologies, Inc.) which has similar efficacy to PL9643 in dry eye models. PL8331 showed optimal behavior at 0.1 μg/mL followed closely by 1.0 μg/mL. Activity for both agonists was lower at 10 μg/mL. PL8331 is 10 times more potent at mouse MC1R *in vitro* than at human MC1R, so the 1 μg/mL dose for PL9643 was chosen for humans to be on the higher side of effective concentrations in view of the lower potency of PL9643 (Palatin Technologies, Inc., data on file).

**FIG. 1.**
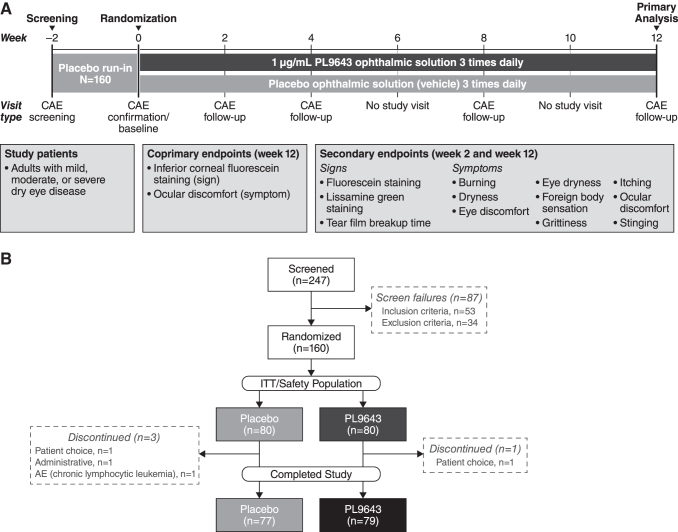
**(A)** Study design. **(B)** Patient disposition. AE, adverse event; CAE, controlled adverse environment; ITT, intent-to-treat.

Patients received single-use blow-fill-seal containers and were instructed to self-administer the ophthalmic solution bilaterally. Randomization was performed by an interactive web response system in which each qualifying patient was given a 4-digit randomization number at the end of baseline (day 1) visit. Patients, sponsor, and the contract research organization were all masked to treatment assignment. A CAE occurred at all visits (baseline and weeks 2, 4, 8, and 12), with pre-CAE, during CAE (symptoms only), and post-CAE assessments of ocular signs and symptoms (Fig. 1A). For some patients, post-CAE assessments were not conducted because of COVID-19 (*n* = 9 PL9643, *n* = 9 placebo). On clinic visit days, dosing was done in the clinic after the post-CAE assessments, but before discharging the patient.

### Patients

Enrolled patients were adults with mild, moderate, or severe DED. Major selection criteria are shown in Table 1. The results were analyzed for the total intent-to-treat (ITT) population of enrolled patients and for those patients who had moderate or severe DED and those with mild disease. The moderate or severe subgroup consisted of those patients who at screening had a duration of dry eye ≥5 years, inferior corneal staining >1 (pre-CAE), and eye discomfort (symptom) on the visual analog scale (VAS) ≥25 (pre-CAE). The mild subgroup consisted of the subgroup of patients who were in the overall ITT population but were not included in the moderate or severe subgroup. These mild patients consisted of those whose duration of DED was 6 months to <5 years, had inferior corneal staining ≤1, and VAS-rated eye discomfort of <25 (pre-CAE).

**Table 1.** Major Patient Selection Criteria

Inclusion ≥18 years of age Reported history of dry eye and had used or desired to use eye drops to treat the condition for ≥6 months before the first treatment visit On treatment visits 1 and 2 patients had to have ○ BCVA of ≥0.7 logMAR (Snellen equivalent score of ≥20/100) ○ Score of ≥2 on the Ora Calibra^®^ Ocular Discomfort and 4-Symptom Questionnaire in ≥1 of the dry eye symptoms ○ Schirmer's test score of ≤10 and ≥1 mm^a^ ○ Corneal fluorescein staining score of ≥2 in any corneal region (inferior, central, or superior) according to the Ora Calibra Corneal and Conjunctival Staining Scale for Grading of Fluorescein Staining in ≥1 eye^a^ ○ Conjunctival redness score ≥1 according to the Ora Calibra Conjunctival Redness for Dry Eye Scale in ≥1 eye (2-week visit pre-CAE)^a^ Demonstrated response to CAE at baseline and 2-week visit in the same eye(s)^a^ ○ ≥1 point increase in fluorescein staining in the inferior region in ≥1 eye after CAE exposure ○ Ocular Discomfort score ≥3 at ≥2 time points in ≥1 eye during CAE exposure Exclusion Clinically significant slit-lamp findings at screening, including active blepharitis, meibomian gland dysfunction, lid margin inflammation, or active ocular allergies that required therapeutic treatment or would interfere with study Been diagnosed with an ongoing ocular infection or active ocular inflammation Worn contact lenses within 7 days of screening or planned to wear LASIK surgery performed within 12 months, ocular and/or lid surgeries in past 6 months or planned during the study Laser procedures in the previous 3 months Use of cyclosporine or lifitegrast ophthalmic solutions within 60 days of screening Use of or anticipated use of temporary punctal plugs during study that were not stable within 30 days of screening Unable to discontinue topical ophthalmic prescriptions for the duration of the trial

^a^ At least 1 eye (same eye) had to satisfy all these criteria.

BCVA, best-corrected visual acuity; CAE, controlled adverse environment; logMAR, logarithm of the minimum angle of resolution.

### Efficacy endpoints

The coprimary endpoints were the degree of inferior corneal fluorescein staining and ocular discomfort after 12 weeks (day 85) versus that shown at baseline (day 0), pre- to post-CAE change.

Corneal fluorescein staining was measured by the Ora Calibra^®^ Corneal and Conjunctival Staining Scale (Ora Calibra, Andover, MA),^18^ a 5-point scale from 0 (no staining) to 4 (severe staining) with 0.5-U increments. Ocular discomfort was measured by the Ora Calibra Ocular Discomfort Scale^19,20^ which ranges from 0 to 4, in which 0 = no discomfort, 1 = intermittent awareness, 2 = constant awareness, 3 = intermittent discomfort, and 4 = constant discomfort.

Secondary efficacy measures were changes in additional signs and symptoms of dry eye at all visits (Table 2). Eyes were eligible for analysis if they met all of the inclusion criteria. In the case that both eyes were eligible for analysis, the study eye was the eye with the higher inferior corneal fluorescein staining at baseline (week 1/day 1) pre-CAE to post-CAE change. If inferior corneal fluorescein staining pre-CAE to post-CAE change was equivalent in both eyes, then the study eye was the eye with the higher ocular discomfort at day 1 (visit 2) pre-CAE. If ocular discomfort was equivalent in both eyes, then the right eye was selected as the study eye.

**Table 2.** Secondary Endpoints Assessed in the Study

Secondary endpoint	Method/scale	Scoring
Signs		
Corneal damage	Fluorescein staining^a^	5-point scale (0.5 U increments) grade 0 = none, 4 = severe
	Lissamine Green staining^a^	5-point scale (0.5 U increments) grade 0 = none, 4 = severe
Conjunctival redness	Ora Calibra Conjunctival Redness Scale^21^	5-point scale (0.5-U increments); 0 = none, 4 = severe
Tear production	Schirmer's test (unanesthetized)	—
Tear film breakup	—	For each eye, 2 measurements were recorded and averaged unless the 2 measurements were >2 s apart and were each <10 s, in which case, a 3rd measurement was taken and the 2 closest of the 3 were averaged and used for analyses. Analyses were for the study eye only
Symptoms		
Ocular discomfort	Ora Calibra Ocular Discomfort Scale^19,20^	5-point scale; 0 = no discomfort, 1 = intermittent awareness, 2 = constant awareness, 3 = intermittent discomfort, 4 = constant discomfort
	Ora Calibra Ocular Discomfort and 4-Symptom Questionnaire^19,20^	6-point scale; 0 = no pain, 5 = the worst pain. 5 symptoms are graded separately: overall discomfort, burning, dryness, grittiness, and stinging
	Ocular Surface Disease Index^22^	Scale of 0–100, with higher scores representing greater disability
	Visual analog scale: Symptoms assessed were burning/stinging, itching, foreign body sensation, eye discomfort, blurred vision, eye dryness, photophobia, and pain	Scored by asking patients to rate each ocular symptom due to ocular dryness by placing a mark on a 100-mm horizontal line to indicate the level of discomfort; 0 = no discomfort, 100 = maximal discomfort
	Ora Calibra Drop Comfort Scale and Questionnaire^21^^,^^b^	11-point scale (0–10); 0 = very comfortable, 10 = very uncomfortable. Assessed immediately and at 1 and 2 min after dosing

^a^ By region (central, superior, inferior, temporal, nasal, corneal sum, conjunctival sum, and total staining). Corneal sum was the sum of the central, superior, and inferior regions (range 0–12). Conjunctival sum was the sum of the temporal and nasal regions (range 0–6). Total eye score was the sum of all 5 regions (range 0–20). Analyses were for the study eye only.

^b^ Assessed at baseline only.

### Safety

Visual acuity was assessed pre-CAE at all visits (screening; baseline; and weeks 2, 4, 8, and 12). Slit-lamp evaluation was assessed at all visits both pre- and post-CAE. Intraocular pressure and dilated fundus examination was performed post-CAE at screening and week 12. Adverse events (AEs) were recorded throughout the study at all visits.

### Sample size

This study was expected to enroll 75 patients in each of the 2 treatment arms, for a total of 150 randomized patients. Assuming a 10% dropout rate, 67 patients per group were expected to complete the study. Assuming a common standard deviation (SD) in the change from baseline for the pre-CAE to post-CAE change in inferior corneal fluorescein staining of 0.80 U, a sample size of 67 patients per treatment group could detect a mean difference of 0.39 U between the active treatment group and the placebo group at a significance level of 0.05 with 80% power. A sample size of 67 patients per treatment group could detect a mean difference of 0.49 U in the change from baseline for the pre-CAE ocular discomfort as assessed by the Ora Calibra Ocular Discomfort Scale with 80% power, assuming an SD of 1.00 U. The power for both the sign and symptom endpoints was 64%, assuming independence between the endpoints. Although sample size calculations were based on completed patients, primary analysis for sign and symptom primary endpoints were conducted on the ITT population using Markov chain Monte Carlo imputation.

### Statistics

For each endpoint, change from baseline was calculated for each patient as week 12 (day 85) − baseline (day 1), such that a positive difference indicated a worsening of the dry eye sign or symptom. In addition, comparisons between active treatment and placebo were calculated as active −placebo, such that a negative result indicated a better score for the active treatment. Pre- to post-CAE change was calculated as post-CAE − pre-CAE. For the analysis of the endpoints, patients without a recorded value at the final day 85 visit had their observations imputed using Markov chain Monte Carlo imputation under the assumption of a missing-at-random mechanism using the ITT population. Inferior corneal fluorescein staining was summarized at week 12 pre-CAE to post-CAE change by treatment groups using continuous descriptive statistics. Analysis of the change from baseline used an analysis of covariance model adjusted for baseline inferior corneal fluorescein staining pre-CAE to post-CAE change and with treatment group as the explanatory variable. Changes from baseline for each treatment group in inferior corneal fluorescein staining were compared between 1 μg/mL PL9643 ophthalmic solution and placebo. Ocular discomfort was summarized at week 12 pre-CAE by treatment groups using continuous descriptive statistics. The type I error for secondary endpoints was not adjusted for multiplicity. Therefore, statistical significance for these endpoints, which was determined based on a 2-sided *α* = 0.05, should be viewed as informative. *P* values in the text are labeled as “nominal” *P* values to indicate that they are not corrected for multiplicity. All statistical tests were 2-sided with a significance level of 0.05 unless otherwise specified. Confidence intervals (CIs) for differences between treatment groups were 2-sided at 95% confidence. Statistical programming and analyses were performed using Statistical Analysis Software^®^ (SAS, Cary, NC) version 9.4.

## Results

### Baseline characteristics and demographics

The study was started on February 15, 2020, and completed on October 5, 2020. A total of 160 patients were randomized: 80 in the PL9643 group and 80 in the placebo group. A total of 156 patients (97.5%) completed the study: 79 (98.8%) in the PL9643 group and 77 (96.3%) in the placebo group. A total of 4 patients (2.5%) were discontinued from this study: 1 (1.3%) in the PL9643 group and 3 (3.8%) in the placebo group (Fig. 1B). A patient in the placebo group discontinued due to a serious AE of chronic lymphocytic leukemia (considered unrelated to the study drug), and the patient in the PL9643 group withdrew due to patient choice.

The demographic and baseline characteristics of the entire enrolled population are shown in Table 3. The subgroup analyses were performed on patients who were classified as having moderate or severe dry eye (*n* = 53) and also on those with mild DED (*n* = 107). Baseline demographics of the patients with moderate and severe disease were balanced across the 2 groups. Median age was 69 (range, 51–84) years for the placebo group and 69.5 (range, 51–80) years for the PL9643 group. All patients had the same eye color reported for both their right eye and left eye. Patients in the overall population were exposed to study drug for a mean (SD) of 82.9 (7.69) days and a median exposure of 84 days (range, 16–88 days). Mean (SD) exposure to study drug was similar between the overall treatment groups: 82.6 (7.6) days in placebo group and 83.2 (7.8) days in PL9643 group.

**Table 3.** Patient Characteristics for the Overall Intent-to-Treat Population

	Placebo (*n* = 80)	PL9643 (*n* = 80)	All patients (*n* = 160)
Age, years			
Mean (SD)	63.8 (12.38)	62.0 (12.55)	62.9 (12.46)
Median (minimum, maximum)	65.0 (26, 84)	62.5 (25, 87)	64.0 (25, 87)
Sex, *n* (%)			
Male	27 (33.8)	21 (26.3)	48 (30.0)
Female	53 (66.3)	59 (73.8)	112 (70.0)
Ethnicity, *n* (%)			
Hispanic or Latino	3 (3.8)	1 (1.3)	4 (2.5)
Not Hispanic or Latino	77 (96.3)	79 (98.8)	156 (97.5)
Race, *n* (%)			
Asian	1 (1.3)	0	1 (0.6)
Black or African American	17 (21.3)	16 (20.0)	33 (20.6)
White	62 (77.5)	63 (78.8)	125 (78.1)
Multiple	0	1 (1.3)	1 (0.6)

SD, standard deviation.

### Efficacy in the overall (ITT) populations

For the overall population of patients with mild to severe DED, for inferior corneal fluorescein staining the PL9643 group showed better improvement than the placebo group, but did not demonstrate a statistically significant difference in mean change from baseline at week 12 compared to the placebo group (pre-CAE to post-CAE) with a PL9643 versus placebo least squares (LS) mean (95% CI) change of −0.11 (−0.38 to 0.16; *P* = 0.4363) (Table 4). There was no improvement versus placebo for ocular discomfort (PL9643 vs. placebo LS mean 0 [−0.3 to 0.4]; *P* = 0.8360). For the secondary endpoints, the PL9643 group did not demonstrate a significant difference in mean change from baseline at any visit or time point compared to the placebo group in the ITT population with observed data.

**Table 4.** Primary Efficacy Endpoints for the Intent-to-Treat Population

Visit	Placebo (*n* = 80)	PL9643 (*n* = 80)
Inferior corneal staining		
Baseline		
Mean (SD)	1.45 (0.537)	1.42 (0.524)
95% CI	1.33 to 1.57	1.30 to 1.54
Week 12		
Mean (SD)	0.77 (0.885)	0.65 (0.895)
95% CI	0.58 to 0.96	0.46 to 0.85
Change from baseline to week 12		
LS mean (SE)	–0.67 (0.100)	–0.78 (0.098)
95% CI	–0.86 to −0.47	–0.97 to −0.58
PL9643 vs. placebo		
LS mean difference (SE)	–0.11 (0.139)	
95% CI	–0.38 to 0.16 (*P* = 0.4363)	
Ocular discomfort		
Baseline		
Mean (SD)	2.4 (0.93)	–0.6 (1.18)
95% CI	2.2 to 2.6	–0.8 to −0.3
Week 12		
Mean (SD)	1.8 (1.18)	–0.5 (0.12)
95% CI	1.6 to 2.1	–0.8 to −0.3
Change from baseline to week 12		
LS mean (SE)	–0.6 (0.12)	–0.5 (0.12)
95% CI	–0.8 to −0.3	–0.8 to −0.3
PL9643 vs. placebo		
LS mean difference (SE)	0 (0.16)	
95% CI	–0.3 to 0.4 (*P* = 0.8360)	

95% CI, 95% confidence interval; LS, least squares; SE, standard error.

### Efficacy endpoints in the moderate or severe dry eye subpopulation

The *post hoc* analysis of the moderate or severe dry eye subpopulation, in contrast to the ITT population, showed nominal statistical significance (*P* < 0.05 vs. placebo) at week 2 and 12 for multiple signs and symptoms of DED compared with the ophthalmic solution placebo.

#### Fluorescein staining

**FIG. 2.**
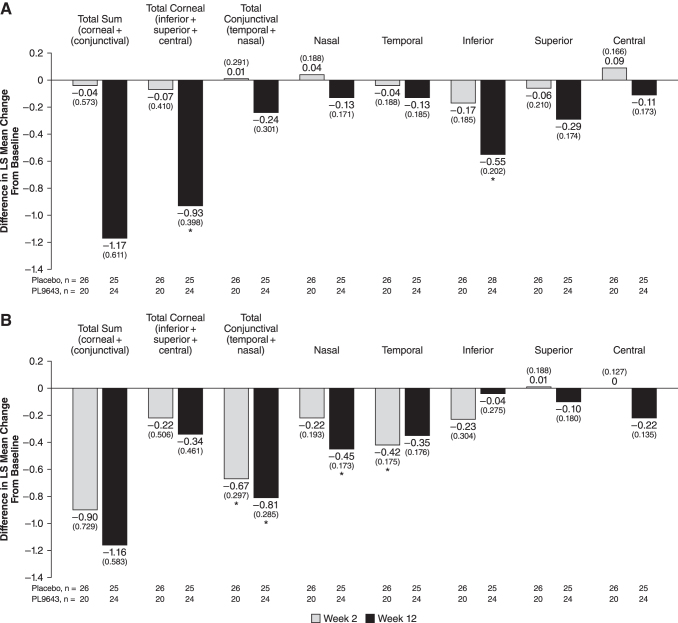
Difference between PL9643 and placebo at weeks 2 and 12 for the population with moderate to severe DED for corneal and conjunctival fluorescein staining **(A)** and corneal and conjunctival Lissamine Green staining **(B)**. Note: Treatment difference was LS (SE) change from pre-CAE baseline to post-CAE for each treatment LS mean change from baseline. Fluorescein and Lissamine Green staining were measured with the Ora Calibra Corneal and Conjunctival Staining Scale. *Nominal *P* < 0.05 versus placebo through analysis of covariance. Note: the type I error for secondary endpoints was not adjusted for multiplicity. Therefore, statistical significance for these endpoints should be viewed as hypothesis generating and not as statistical evidence. CAE, controlled adverse environment; DED, dry eye disease; LS, least squares; SE, standard error.

Improvement from baseline was observed for the PL9643 group compared with the placebo group for inferior corneal fluorescein staining at week 12 (day 85, change from pre-CAE to post-CAE) with a LS mean (standard error [SE]) change of −0.92 (0.144) versus −0.38 (0.142), respectively, an LS mean treatment difference of −0.55 (0.202; nominal *P* = 0.0097). PL9643 treatment also demonstrated an improvement in total corneal (inferior, superior, and central) fluorescein staining at week 12 with an LS mean (SE) change of −1.41 (0.284) compared with −0.48 (0.278) for placebo, an LS mean (SE) treatment difference of −0.93 (0.398; nominal *P* = 0.0242) (Fig. 2A and Supplementary Table S1).

In addition, PL9643 treatment demonstrated some improvement over placebo in mean change from baseline at week 12 in superior corneal and conjunctival fluorescein staining (LS mean [standard error of the mean, SEM] difference, −0.29 [0.174]; nominal *P* = 0.0977) and total sum of all 5 regions (LS mean [SEM] difference, −1.17 [0.611]; nominal *P* = 0.0606).

#### Lissamine Green staining

PL9643 treatment demonstrated an improvement over placebo in mean change from baseline in nasal staining at week 12 (change from pre-CAE to post-CAE) with an LS mean (SE) change of −0.36 (0.124) versus +0.09 (0.121), respectively, an LS mean treatment difference of −0.45 (0.173; nominal *P* = 0.0118). PL9643 treatment also demonstrated a significant improvement in conjunctival sum (temporal and nasal) staining at week 12 with an LS mean (SEM) change of −0.56 (0.204) compared with +0.25 (0.199) for placebo, an LS mean (SEM) treatment difference of −0.81 (0.285; nominal *P* = 0.0068). PL9643 treatment also demonstrated an improvement in conjunctival sum (temporal and nasal) staining at week 2 with an LS mean (SEM) change of −0.89 (0.223) compared with −0.22 (0.196) for placebo, an LS mean (SEM) treatment difference of −0.67 (0.297; nominal *P* = 0.0292) (Fig. 2B, Supplementary Table S2). In addition, PL9643 treatment demonstrated considerable improvement over placebo in mean change from baseline in total sum (of all 5 regions) Lissamine Green staining at week 12 of −1.16 (0.583, *P* = 0.0533).

#### Conjunctival redness

**FIG. 3.**
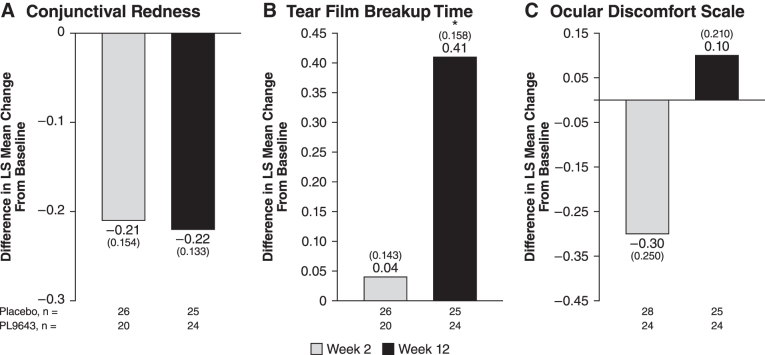
Difference between PL9643 and placebo at weeks 2 and 12 for the population with moderate to severe DED for conjunctival redness **(A)**, tear film breakup time **(B)**, and ocular discomfort **(C)**. Treatment difference was LS mean (SE) change from baseline post-CAE for **(A, B)** and was pre-CAE for **(C)**. Conjunctival redness was measured by the Ora Calibra Conjunctival Redness Scale; ocular discomfort was measured by the Ora Calibra Ocular Discomfort Scale. *Nominal *P* < 0.05 versus placebo through analysis of covariance. The type I error for secondary endpoints was not adjusted for multiplicity. Therefore, statistical significance for these endpoints should be viewed as hypothesis generating and not as statistical evidence. CAE, controlled adverse environment; DED, dry eye disease; LS, least squares.

PL9643 treatment showed no significant improvement over placebo at either week 2 or 12 for conjunctival redness post-CAE as measured by the Ora Calibra Conjunctival Redness Scale (Fig. 3A and Supplementary Table S3).

#### Tear film breakup time

PL9643 treatment demonstrated improvement over placebo in LS mean change from baseline at week 12 post-CAE, with an LS mean (SE) treatment difference of +0.41 (0.158) seconds (nominal *P* = 0.0137) (Fig. 3B).

#### Symptoms: Ora Calibra Ocular Discomfort Scale

As measured by the Ora Calibra Ocular Discomfort Scale, which is geared toward measuring of frequency of discomfort, PL9643 treatment showed mean improvement over placebo at week 12 pre-CAE of −0.4 (0.27; nominal *P* = 0.1302) in the moderate or severe dry eye subpopulation although these differences were not significant (Fig. 3C). During the 90-min exposure to the CAE, PL9643 treatment demonstrated an improvement (nominally *P* < 0.05) or a trend toward improvement over placebo in mean change from baseline. The LS mean change (−0.3; nominal *P* < 0.05) from baseline at 20 min during the CAE challenge showed less discomfort versus placebo as did the 25-min difference (−0.3; nominal *P* = 0.0684). However, at the other measured time points, PL9643 scored showed no differences with nominal *P* values >0.05 from placebo.

#### Symptoms: Ora Calibra Ocular Discomfort and 4-Symptom Questionnaire

**FIG. 4.**
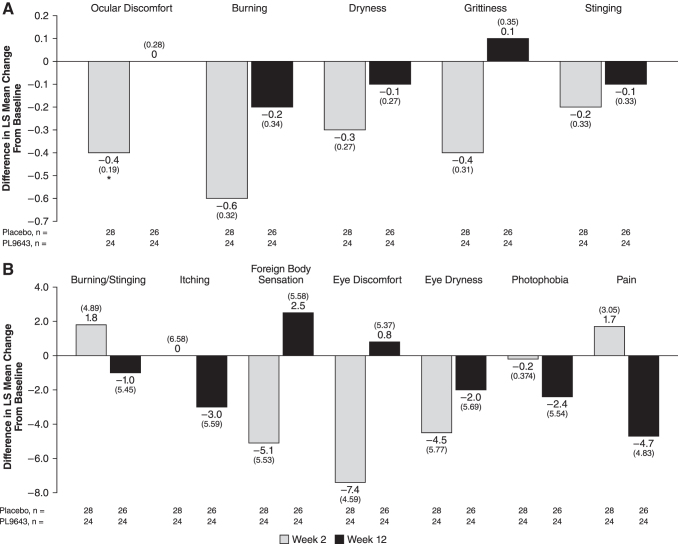
Difference between PL9643 and placebo at weeks 2 and 12 for the population with moderate to severe DED. Ora Calibra Ocular Discomfort and 4-Symptom Questionnaire **(A)** and Visual Analog Scale **(B)**. Treatment difference was LS mean (SE) change from pre-CAE baseline. *Nominal *P* < 0.05 versus placebo through analysis of covariance. Note: the type I error for secondary endpoints was not adjusted for multiplicity. Therefore, statistical significance for these endpoints should be viewed as hypothesis generating and not as statistical evidence. CAE, controlled adverse environment; DED, dry eye disease; LS, least squares.

Using the Ora Calibra Ocular Discomfort and 4-Symptom Questionnaire, a measure of discomfort severity, PL9643 treatment demonstrated improvement over placebo in mean change from baseline at week 2 in ocular discomfort (mean treatment difference −0.4 (0.19); nominal *P* = 0.0227), although there was no important difference at week 12. Burning at week 2 showed a larger treatment difference in favor of PL9643, with a mean difference from placebo of −0.6 (0.32; nominal *P* = 0.0624). Other symptoms measured by the questionnaire (dryness, stinging, and grittiness) all showed a numerically superior (but nonsignificant) difference compared with placebo throughout the duration of the study, except for grittiness at week 12 (Fig. 4A and Supplementary Table S4).

#### Symptoms: VAS

PL9643 treatment showed nonsignificant numerical improvement over placebo in LS mean change from baseline at week 2 for the VAS symptoms of itching (−1), foreign body sensation (−5.1), eye discomfort (−7.4), and eye dryness (−4.5). The treatment effect of PL9643 was generally maintained throughout the study for symptoms of eye discomfort, eye dryness, and itching from week 2 to 12. The greatest LS mean treatment differences at week 12 were observed for pain (−4.7) and itching (−3.0) (Fig. 4B and Supplementary Table S5).

### Efficacy in the mild DED subpopulation

The subgroup with mild disease showed no significant differences in mean change from baseline compared to placebo. Inferior corneal staining, total corneal staining, conjunctival redness, ocular discomfort, and eye discomfort all showed improvement with PL9634 administration, but none was superior to placebo. Mean tear film breakup time stayed level over the duration of the study but was not better than placebo at any visit.

### Drop comfort (ITT population)

Drop comfort was assessed for each eye immediately and at 1 and 2 min following initial dosing using the Ora Calibra Drop Comfort Scale at baseline. Descriptions of drop comfort using the Ora Calibra Drop Comfort Scale were assessed at 3 min after initial dosing. On this questionnaire, patients were asked to choose 3 words that best described how each eye drop felt in both of their eyes. The positive responses were comfortable, cool, refreshing, smooth, and soothing. The negative responses included burning, filmy, stinging, sticky, thick, gritty, and irritating. Patients may have also selected “other” and written in a different response, which may have been either positive or a negative.

Responses to the Ora Calibra Drop Comfort Scale at baseline between the treatment groups were comparable, with 67 (83.8%) patients in the PL9643 group and 66 (82.5%) patients in the placebo group reporting ≥1 positive response and 31 (38.8%) patients in the PL9643 group and 29 (36.6%) patients in the placebo group reporting ≥1 negative response. The most common positive responses in the PL9643 group were “cool” and “refreshing” (*n* = 41 each [51.3%]), compared with 44 (55.0%) and 36 (45.0%) patients in the placebo group, respectively. The most common negative response in the PL9643 group was “irritating,” (*n* = 14 [17.5%]), compared with 10 (12.5%) patients in the placebo group.

### Safety and tolerability (ITT population)

In the overall ITT/safety population (mild, moderate, and severe dry eye), more patients experienced more AEs and more treatment-emergent AEs (TEAEs) in the placebo group than in the PL9643 group (AEs: 22 [27.5%] vs. 16 [20.0%]; TEAEs: 20 [25.0%] vs. 13 [16.3%]) (Table 5). For a composite of TEAEs of particular interest for an ophthalmic solution: instillation pain, instillation site irritation, reduction in visual acuity, and eye pruritus there were 7 patients (8.8%) in the placebo group and none in the PL9643 group (difference PL9643-placebo *P* = 0.0136, Fisher's exact test). The most common ocular TEAE was instillation site pain in a total of 5 (3.8%) patients: all 5 (6.3%) patients were in the placebo group. These incidences of instillation site pain were considered to be treatment-related (categorized as definitely related, probably related, or possibly related to study drug) and trended toward significance in favor of fewer patients having pain in the PL9643 arm (*P* = 0.0586). One ocular TEAE of chalazion was reported in the PL9643 group. Nonocular TEAEs were reported by a total of 24 (15.0%) patients, 12 (15.0%) patients in the PL9643 group and 12 (15.0%) patients in the placebo group. The most frequent nonocular TEAEs were in the infections and infestation system organ class, experienced by 6 (3.8%) of patients overall, 2 (2.5%) in the placebo and 4 (5.0%) in the PL9643 groups. All other nonocular TEAEs were reported in less than 3% of patients.

**Table 5.** Adverse Events by Severity and Relation to Treatment (Intent-to-Treat/Safety Population)

	Placebo (*n* = 80)		PL9643 (*n* = 80)		All patients (*n* = 160)	
	Patients,* n *(%)	Events,* n*	Patients,* n *(%)	Events,* n*	Patients,* n *(%)	Events,* n*
AEs	22 (27.5)	31	16 (20.0)	19	38 (23.8)	50
TEAEs	20 (25.0)	23	13 (16.3)	16	33 (20.6)	39
Ocular	8 (10.0)	8	1 (1.3)	1	9 (5.6)	9
Nonocular	12 (15.0)	15	12 (15.0)	15	24 (15.0)	30
TEAE severity						
Mild	15 (18.8)	17	10 (12.5)	13	25 (15.6)	30
Moderate	3 (3.8)	4	3 (3.8)	3	6 (3.8)	7
Severe	2 (2.5)	2	0	0	2 (1.3)	2
TEAE relationship to study drug						
Definitely	4 (5.0)	4	0	0	4 (2.5)	4
Probably	2 (2.5)	2	0	0	2 (1.3)	2
Possibly	2 (2.5)	2	1 (1.3)	1	3 (1.9)	3
Unlikely	0	0	1 (1.3)	1	1 (0.6)	1
Not related	12 (15.0)	15	11 (13.8)	14	23 (14.4)	29
TEAEs causing premature treatment discontinuation	1 (1.3)	1	0	0	1 (0.6)	1
Treatment-emergent SAEs	2 (2.5)	2	1 (1.3)	1	3 (1.9)	3
Ocular treatment-related TEAEs^a^	7 (8.8)	7	0	0	7 (4.4)	7
Instillation site pain	5 (6.3)	5	0	0	5 (3.8)	5
Instillation site pruritus	1 (1.3)	1	0	0	1 (0.6)	1
Instillation site irritation	0	0	0	0	0	0
Visual acuity reduced	1 (1.3)	1	0	0	1 (0.6)	1

^a^ Treatment-related TEAEs are those categorized as definite, probably, and possibly related.

AE, adverse event; SAE, serious adverse event; TEAE, treatment-emergent adverse event.

A total of 3 (1.9%) patients reported 3 treatment-emergent serious AEs: 1 (1.3%) patient in the PL9643 group and 2 (2.5%) patients in the placebo group. The 2 serious AEs in the placebo group were classified as severe (chronic lymphocytic leukemia, deep vein thrombosis), and the serious AE in the single patient in the PL9643 group was reported as moderate (lung adenocarcinoma). No treatment-emergent serious AEs were considered related to the study drug. No clinically significant effects on visual acuity, slit-lamp biomicroscopy, intraocular pressure, or dilated fundoscopy by PL9643 were observed.

## Discussion

This study investigated the effect of an ophthalmic solution of the melanocortin MCR pan-agonist PL9643 on patients with DED over a 12-week period. The study explored a range of signs and symptoms of DED using commonly used measures, including the use of the CAE model. The prospective hypothesis that, in the ITT population of patients with mild to severe DED, PL9643 treatment would show statistically superior efficacy to placebo (pre-CAE to post-CAE) in inferior corneal fluorescein staining and ocular discomfort at week 12 was not supported. The ITT PL9643 group also did not demonstrate a significant difference from the placebo group for the secondary endpoints.

In the *post hoc* analysis subgroup of patients with moderate or severe DED (duration of dry eye ≥5 years, inferior corneal staining >1, and eye discomfort on the VAS ≥25), ophthalmic PL9643 treatment demonstrated either nominally significant (*P* < 0.05) or trending (*P* < 0.1) improvement over the active control of artificial tears in mean change from baseline at week 12/day 85 in several sign endpoints, including fluorescein staining in inferior, superior, corneal sum, and total sum regions; Lissamine Green staining in temporal, nasal, conjunctival sum, and total sum regions; and tear film breakup time. Conjunctival redness also showed improvement at week 12/day 85, although not statistically significant, in favor of PL9643. In the subgroup of patients with mild disease, the magnitude of the effect against the artificial tears placebo was not discernible.

Responses to the Ora Calibra Drop Comfort Scale in the overall ITT population were comparable for the PL9643 group compared with the placebo group, and PL9643-treated patients experienced no AEs of pain on instillation, in contrast to the 3.1% observed in the placebo group. PL9643 was well tolerated, with a safety profile trending toward superiority over placebo in the ITT population. No treatment-emergent serious AEs were considered related to the study drug, and no patients in the PL9643 group withdrew from the study due to AEs. PL9643 also showed no effects on other aspects of vision (visual acuity, slit-lamp biomicroscopy, intraocular pressure, or dilated fundoscopy).

A major limitation of this study is that the results of the secondary endpoints have not been adjusted for multiplicity, so that changes in these endpoints are hypothesis generating only. Further studies with larger sample sizes will be able to address the statistical significance of these changes. The absence of significant improvements in the mild subpopulation may be a consequence of the small sample size. In the future, tests evaluating ocular surface inflammation such as matrix metalloproteinase 9 concentration (or other pro-inflammatory markers) in the tear film offer the potential to aid the evaluation of efficacy.

The purpose of our initial clinical investigation was to identify patients that had the greatest response to PL9643. Although we did not see statistical significance for the overall ITT population, we were able to identify a responsive subpopulation which could be valuable for future clinical studies. In the subpopulation of moderate to severe patients (*n* = 53), PL9643 achieved nominal statistical significance (*P* < 0.05 vs. placebo) at week 2 and 12 for multiple signs and symptoms of DED compared with the ophthalmic solution placebo. PL9643 demonstrated good ocular safety and tolerability with no drug-related AEs, no drug-related discontinuations, and with high ocular comfort. These hypothesis-generating efficacy results and lack of tolerability issues suggest that PL9643 could be a valuable option as a novel therapeutic for treating DED and supports its continued development.

## Supplementary Material

Supplemental data

Supplemental data

Supplemental data

Supplemental data

Supplemental data
