# Supplementary material for: Efficacy and Safety of the Melanocortin Pan-Agonist PL9643 in a Phase 2 Study of Patients with Dry Eye Disease
Source: J Ocul Pharmacol Ther. 2023 Nov 2;39(9):600–10. doi: 10.1089/jop.2023.0056 (PMC10654643; doi:10.1089/jop.2023.0056)
Supplement: Supplemental data [file Suppl_TableS1.docx]

**Supplemental Table 1.** Treatment difference (pre-CAE to post-CAE) between PL9643 and placebo at weeks 2 (day 15) and 12 (day 85) for the population with moderate to severe DED for corneal and conjunctival fluorescein staining

| **Parameter** | **Visit (Day)** | **Treatment** | **N** | **LS Mean** | **95% CI** | **SE** | ***P*-Value (WRS)** | ***P*-Value (ANCOVA)** | **Difference**  **(PL9643 minus Vehicle/Placebo)** | **SE** |
| --- | --- | --- | --- | --- | --- | --- | --- | --- | --- | --- |
| Total corneal (inferior + superior + central) | 15 | Placebo | 26 | -0.99 | -1.53, -0.45 | 0.269 | 0.8502 | 0.8630 | -0.07 | 0.410 |
|  | 15 | PL9643 | 20 | -1.06 | -1.68, -0.44 | 0.307 |  |  |  |  |
|  | 85 | Placebo | 25 | -0.48 | -1.04, 0.07 | 0.278 | 0.0555 | 0.0242 | -0.93 | 0.398 |
|  | 85 | PL9643 | 24 | -1.41 | -1.98, -0.84 | 0.284 |  |  |  |  |
| Total Sum (corneal + conjunctival) | 15 | Placebo | 26 | -1.56 | -2.32, -0.80 | 0.375 | 0.5072 | 0.9460 | -0.04 | 0.573 |
|  | 15 | PL9643 | 20 | -1.6 | -2.46, -0.73 | 0.428 |  |  |  |  |
|  | 85 | Placebo | 25 | -0.62 | -1.48, 0.24 | 0.426 | 0.1756 | 0.0606 | -1.17 | 0.611 |
|  | 85 | PL9643 | 24 | -1.79 | -2.67, -0.92 | 0.435 |  |  |  |  |
| Total conjunctival (temporal + nasal) | 15 | Placebo | 26 | -0.56 | -0.94, -0.17 | 0.191 | 0.2779 | 0.9687 | 0.01 | 0.291 |
|  | 15 | PL9643 | 20 | -0.55 | -0.99, -0.11 | 0.218 |  |  |  |  |
|  | 85 | Placebo | 25 | -0.14 | -0.56, 0.29 | 0.211 | 0.8878 | 0.4255 | -0.24 | 0.301 |
|  | 85 | PL9643 | 24 | -0.38 | -0.81. 0.05 | 0.215 |  |  |  |  |
| Nasal | 15 | Placebo | 26 | -0.3 | -0.55, -0.05 | 0.124 | 0.4370 | 0.8240 | 0.04 | 0.188 |
|  | 15 | PL9643 | 20 | -0.26 | -0.54, 0.03 | 0.142 |  |  |  |  |
|  | 85 | Placebo | 25 | -0.11 | -0.35, 0.13 | 0.120 | 0.7601 | 0.4365 | -0.13 | 0.171 |
|  | 85 | PL9643 | 24 | -0.24 | -0.49, 0.00 | 0.122 |  |  |  |  |
| Temporal | 15 | Placebo | 26 | -0.26 | -0.46, -0.05 | 0.101 | 0.5351 | 0.8108 | -0.04 | 0.154 |
|  | 15 | PL9643 | 20 | -0.29 | -0.52, -0.06 | 0.115 |  |  |  |  |
|  | 85 | Placebo | 25 | -0.02 | -0.27, 0.24 | 0.128 | 0.8792 | 0.4735 | -0.13 | 0.185 |
|  | 85 | PL9643 | 24 | -0.15 | -0.41, 0.11 | 0.131 |  |  |  |  |
| Inferior | 15 | Placebo | 26 | -0.41 | -0.66, -0.17 | 0.121 | 0.4307 | 0.4307 | -0.17 | 0.185 |
|  | 15 | PL9643 | 20 | -0.59 | -0.87, -0.31 | 0.138 |  |  |  |  |
|  | 85 | Placebo | 28 | -0.38 | -0.66, -0.09 | 0.142 | 0.0161 | 0.0097 | -0.55 | 0.202 |
|  | 85 | PL9643 | 24 | -0.92 | -1.21, -0.63 | 0.144 |  |  |  |  |
| Superior | 15 | Placebo | 26 | -0.27 | -0.55, 0.01 | 0.138 | 0.9373 | 0.7687 | -0.06 | 0.210 |
|  | 15 | PL9643 | 20 | -0.33 | -0.65, -0.01 | 0.158 |  |  |  |  |
|  | 85 | Placebo | 25 | -0.06 | -0.30, 0.19 | 0.122 | 0.4457 | 0.0977 | -0.29 | 0.174 |
|  | 85 | PL9643 | 24 | -0.35 | -0.60, -0.10 | 0.124 |  |  |  |  |
| Central | 15 | Placebo | 26 | -0.28 | -0.50, -0.06 | 0.109 | 0.9909 | 0.5767 | 0.09 | 0.166 |
|  | 15 | PL9643 | 20 | -0.19 | -0.44, 0.07 | 0.125 |  |  |  |  |
|  | 85 | Placebo | 25 | -0.04 | -0.28, 0.21 | 0.121 | 0.8853 | 0.5225 | -0.11 | 0.173 |
|  | 85 | PL9643 | 24 | -0.15 | -0.40, 0.10 | 0.123 |  |  |  |  |

ANCOVA, analysis of covariance; CAE, controlled adverse environment; LS, least squares; SE, standard error, WRS, Wilcoxon rank sum test.
