# Supplementary material for: Efficacy and Safety of the Melanocortin Pan-Agonist PL9643 in a Phase 2 Study of Patients with Dry Eye Disease
Source: J Ocul Pharmacol Ther. 2023 Nov 2;39(9):600–10. doi: 10.1089/jop.2023.0056 (PMC10654643; doi:10.1089/jop.2023.0056)
Supplement: Supplemental data [file Suppl_TableS2.docx]

**Supplemental Table 2.** Treatment difference (pre-CAE to post-CAE) between PL9643 and placebo at weeks 2 (day 15) and 12 (day 85) for the population with moderate to severe DED for corneal and conjunctival lissamine green staining

| **Parameter** | **Visit (Day)** | **Treatment** | **N** | **LS Mean** | **95% CI** | **SE** | ***P*-Value**  **(WRS)** | ***P*-Value (ANCOVA)** | **Difference**  **(PL9643 minus Vehicle/Placebo)** | **SE** |
| --- | --- | --- | --- | --- | --- | --- | --- | --- | --- | --- |
| Total Sum (corneal + conjunctival) | 15 | Placebo | 26 | -0.61 | -1.58, 0.36 | 0.481 | 0.2451 | 0.2264 | -0.90 | 0.729 |
|  | 15 | PL9643 | 20 | -1.51 | -2.61, -0.40 | 0.548 |  |  |  |  |
|  | 85 | Placebo | 25 | -0.29 | -1.11, 0.53 | 0.408 | 0.0743 | 0.0533 | -1.16 | 0.583 |
|  | 85 | PL9643 | 24 | -1.45 | -2.29, -0.61 | 0.416 |  |  |  |  |
| Total corneal (inferior + superior + central) | 15 | Placebo | 26 | -0.39 | -1.06, 0.28 | 0.332 | 0.5879 | 0.6572 | -0.22 | 0.506 |
|  | 15 | PL9643 | 20 | -0.62 | -1.38, 0.15 | 0.379 |  |  |  |  |
|  | 85 | Placebo | 25 | -0.55 | -1.19, 0.10 | 0.322 | 0.2921 | 0.4587 | -0.34 | 0.461 |
|  | 85 | PL9643 | 24 | -0.89 | -1.55, -0.23 | 0.329 |  |  |  |  |
| Total conjunctival (temporal + nasal) | 15 | Placebo | 26 | -0.22 | -0.61, 0.18 | 0.196 | 0.0823 | 0.0292 | -0.67 | 0.297 |
|  | 15 | PL9643 | 20 | -0.89 | -1.34, -0.44 | 0.223 |  |  |  |  |
|  | 85 | Placebo | 25 | 0.25 | -0.15, 0.65 | 0.199 | 0.0242 | 0.0068 | -0.81 | 0.285 |
|  | 85 | PL9643 | 24 | -0.56 | -0.96, -0.15 | 0.204 |  |  |  |  |
| Nasal | 15 | Placebo | 26 | -0.24 | -0.50, 0.02 | 0.127 | 0.4555 | 0.2579 | -0.22 | 0.193 |
|  | 15 | PL9643 | 20 | -0.46 | -0.75, -0.17 | 0.145 |  |  |  |  |
|  | 85 | Placebo | 25 | 0.09 | -0.15, 0.33 | 0.121 | 0.0394 | 0.0118 | -0.45 | 0.173 |
|  | 85 | PL9643 | 24 | -0.36 | -0.61, -0.12 | 0.124 |  |  |  |  |
| Temporal | 15 | Placebo | 26 | 0.01 | -0.22, 0.24 | 0.115 | 0.0286 | 0.0204 | -0.42 | 0.175 |
|  | 15 | PL9643 | 20 | -0.41 | -0.68, -0.15 | 0.132 |  |  |  |  |
|  | 85 | Placebo | 25 | 0.16 | -0.09, 0.41 | 0.123 | 0.0894 | 0.0513 | -0.35 | 0.176 |
|  | 85 | PL9643 | 24 | -0.19 | -0.44, 0.06 | 0.126 |  |  |  |  |
| Inferior | 15 | Placebo | 26 | -0.25 | -0.65, 0.16 | 0.201 | 0.4779 | 0.4607 | -0.23 | 0.304 |
|  | 15 | PL9643 | 20 | -0.48 | -0.94, -0.01 | 0.229 |  |  |  |  |
|  | 85 | Placebo | 25 | -0.54 | -0.93, -0.16 | 0.192 | 0.6156 | 0.8860 | -0.04 | 0.275 |
|  | 85 | PL9643 | 24 | -0.58 | -0.98, -0.19 | 0.196 |  |  |  |  |
| Superior | 15 | Placebo | 26 | -0.08 | -0.33, 0.17 | 0.124 | 0.785 | 0.9607 | 0.01 | 0.188 |
|  | 15 | PL9643 | 20 | -0.07 | -0.36, 0.21 | 0.141 |  |  |  |  |
|  | 85 | Placebo | 25 | -0.02 | -0.28, 0.23 | 0.125 | 0.3607 | 0.5931 | -0.10 | 0.180 |
|  | 85 | PL9643 | 24 | -0.12 | -0.38, 0.14 | 0.128 |  |  |  |  |
| Central | 15 | Placebo | 26 | -0.07 | -0.23, 0.10 | 0.084 | 0.9066 | 0.9978 | 0 | 0.127 |
|  | 15 | PL9643 | 20 | -0.07 | -0.26, 0.13 | 0.096 |  |  |  |  |
|  | 85 | Placebo | 25 | 0.02 | -0.17, 0.21 | 0.094 | 0.2575 | 0.1144 | -0.22 | 0.135 |
|  | 85 | PL9643 | 24 | -0.19 | -0.39, 0.00 | 0.096 |  |  |  |  |

ANCOVA, analysis of covariance; CAE, controlled adverse environment; LS, least squares; SE, standard error, WRS, Wilcoxon rank sum test.
