# Supplementary material for: Efficacy and Safety of the Melanocortin Pan-Agonist PL9643 in a Phase 2 Study of Patients with Dry Eye Disease
Source: J Ocul Pharmacol Ther. 2023 Nov 2;39(9):600–10. doi: 10.1089/jop.2023.0056 (PMC10654643; doi:10.1089/jop.2023.0056)
Supplement: Supplemental data [file Suppl_TableS3.docx]

**Supplemental Table 3.** Difference between PL9643 and placebo at weeks 2 (day 15) and 12 (day 85) for the population with moderate to severe DED for conjunctival redness, tear film breakup time, and ocular discomfort (C). Note: Difference was change from baseline for the post-CAE values for conjunctival redness and tear film breakup time, and change from baseline for the pre-CAE values for ocular discomfort. Conjunctival redness was measured by the Ora Calibra Conjunctival Redness Scale; ocular discomfort was measured by the Ora Calibra Ocular Discomfort Scale

| **Parameter** | **Visit (Day)** | **Treatment** | **N** | **LS Mean** | **95% CI** | **SE** | ***P*-Value**  **(WRS)** | ***P*-Value (ANCOVA)** | **Difference**  **(PL9643 minus Vehicle/Placebo)** | **SE** |
| --- | --- | --- | --- | --- | --- | --- | --- | --- | --- | --- |
| Conjunctival Redness | 15 | Placebo | 26 | 0.16 | -0.05, 0.36 | 0.101 | 0.3599 | 0.1750 | -0.21 | 0.154 |
|  | 15 | PL9643 | 20 | -0.05 | -0.29, 0.18 | 0.115 |  |  |  |  |
|  | 85 | Placebo | 25 | -0.13 | -0.31, 0.06 | 0.093 | 0.2297 | 0.2062 | -0.22 | 0.133 |
|  | 85 | PL9643 | 24 | -0.35 | -0.54, -0.16 | 0.095 |  |  |  |  |
| Tear Film Breakup Time | 15 | Placebo | 26 | 0.13 | -0.05, 0.32 | 0.092 | 0.9385 | 0.7962 | 0.04 | 0.143 |
|  | 15 | PL9643 | 20 | 0.17 | -0.04, 0.38 | 0.106 |  |  |  |  |
|  | 85 | Placebo | 25 | 0.13 | -0.09, 0.35 | 0.110 | 0.2137 | 0.0137 | 0.41 | 0.158 |
|  | 85 | PL9643 | 24 | 0.54 | 0.31, 0.76 | 0.112 |  |  |  |  |
| Ocular Discomfort Scale | 15 | Placebo | 28 | 0.1 | -0.2, 0.5 | 0.170 | 0.2901 | 0.2018 | -0.30 | 0.25 |
|  | 15 | PL9643 | 24 | -0.2 | -0.6, 0.2 | 0.190 |  |  |  |  |
|  | 85 | Placebo | 25 | -0.4 | -0.7, -0.1 | 0.150 | 0.9536 | 0.5765 | 0.10 | 0.21 |
|  | 85 | PL9643 | 24 | -0.2 | -0.6, 0.1 | 0.150 |  |  |  |  |

ANCOVA, analysis of covariance; CAE, controlled adverse environment; LS, least squares; SE, standard error, WRS, Wilcoxon rank sum test.
