# Supplementary material for: Efficacy and Safety of the Melanocortin Pan-Agonist PL9643 in a Phase 2 Study of Patients with Dry Eye Disease
Source: J Ocul Pharmacol Ther. 2023 Nov 2;39(9):600–10. doi: 10.1089/jop.2023.0056 (PMC10654643; doi:10.1089/jop.2023.0056)
Supplement: Supplemental data [file Suppl_TableS4.docx]

**Supplemental Table 4.** Difference (change pre-CAE) between PL9643 and placebo at weeks 2 and 12 for the population with moderate to severe DED. Measured by the Ora Calibra Ocular Discomfort and 4-Symptom Questionnaire

| **Parameter** | **Visit (Day)** | **Treatment** | **N** | **LS Mean** | **95% CI** | **SE** | ***P*-Value**  **(WRS)** | ***P*-Value (ANCOVA)** | **Difference**  **(PL9643 minus Vehicle/Placebo)** | **SE** |
| --- | --- | --- | --- | --- | --- | --- | --- | --- | --- | --- |
| Ocular Discomfort | 15 | Placebo | 28 | 0 | -0.3, 0.2 | 0.130 | 0.0451 | 0.0227 | -0.4 | 0.19 |
|  | 15 | PL9643 | 24 | -0.5 | -0.7, -0.2 | 0.140 |  |  |  |  |
|  | 85 | Placebo | 26 | -0.5 | -0.9, -0.1 | 0.190 | >0.9999 | 0.9114 | 0 | 0.28 |
|  | 85 | PL9643 | 24 | -0.6 | -1.0, -0.1 | 0.200 |  |  |  |  |
| Burning | 15 | Placebo | 28 | 0.1 | -0.3, 0.6 | 0.220 | 0.0671 | 0.0624 | -0.6 | 0.32 |
|  | 15 | PL9643 | 24 | -0.5 | -0.9, 0.0 | 0.230 |  |  |  |  |
|  | 85 | Placebo | 26 | -0.1 | -0.6, 0.3 | 0.240 | 0.5750 | 0.5032 | -0.2 | 0.34 |
|  | 85 | PL9643 | 24 | -0.4 | -0.9, 0.1 | 0.240 |  |  |  |  |
| Dryness | 15 | Placebo | 28 | -0.3 | -0.7, 0.1 | 0.190 | 0.3639 | 0.2674 | -0.3 | 0.27 |
|  | 15 | PL9643 | 24 | -0.6 | -1.0, -0.2 | 0.200 |  |  |  |  |
|  | 85 | Placebo | 26 | -0.4 | -0.8, 0.0 | 0.190 | 0.9505 | 0.6665 | -0.1 | 0.27 |
|  | 85 | PL9643 | 24 | -0.5 | -0.9, -0.1 | 0.190 |  |  |  |  |
| Grittiness | 15 | Placebo | 28 | -0.2 | -0.6, 0.2 | 0.210 | 0.6306 | 0.1818 | -0.4 | 0.31 |
|  | 15 | PL9643 | 24 | -0.6 | -1.1, -0.2 | 0.220 |  |  |  |  |
|  | 85 | Placebo | 26 | -0.5 | -1.0, 0.0 | 0.240 | 0.2536 | 0.7900 | 0.1 | 0.35 |
|  | 85 | PL9643 | 24 | -0.4 | -0.9, 0.1 | 0.250 |  |  |  |  |
| Stinging | 15 | Placebo | 28 | -0.2 | -0.6, 0.2 | 0.200 | 0.8029 | 0.4926 | -0.2 | 0.33 |
|  | 15 | PL9643 | 24 | -0.4 | -0.9, 0.0 | 0.300 |  |  |  |  |
|  | 85 | Placebo | 26 | -0.2 | -0.7, 0.2 | 0.220 | 0.8625 | 0.7557 | -0.1 | 0.33 |
|  | 85 | PL9643 | 24 | -0.3 | -0.7, 0.5 | 0.320 |  |  |  |  |

ANCOVA, analysis of covariance; CAE, controlled adverse environment; LS, least squares; SE, standard error, WRS, Wilcoxon rank sum test.
