# Supplementary material for: Efficacy and Safety of the Melanocortin Pan-Agonist PL9643 in a Phase 2 Study of Patients with Dry Eye Disease
Source: J Ocul Pharmacol Ther. 2023 Nov 2;39(9):600–10. doi: 10.1089/jop.2023.0056 (PMC10654643; doi:10.1089/jop.2023.0056)
Supplement: Supplemental data [file Suppl_TableS5.docx]

**Supplemental Table 5.** Difference (change pre-CAE) between PL9643 and placebo at weeks 2 and 12 for the population with moderate to severe DED. Measured by the Visual Analog Scale

| **Parameter** | **Visit (Day)** | **Treatment** | **N** | **LS Mean** | **95% CI** | **SE** | ***P*-Value**  **(WRS)** | ***P*-Value (ANCOVA)** | **Difference**  **(PL9643 minus Vehicle/Placebo)** | **SE** |
| --- | --- | --- | --- | --- | --- | --- | --- | --- | --- | --- |
| Burning / Stinging | 15 | Placebo | 28 | 0.8 | -5.8, 7.5 | 3.330 | 0.8477 | 0.7195 | 1.8 | 4.89 |
|  | 15 | PL9643 | 24 | 2.6 | -4.6, 9.8 | 3.590 |  |  |  |  |
|  | 85 | Placebo | 26 | 2.6 | -5.0, 10.2 | 3.780 | 0.8769 | 0.8503 | -1.0 | 5.45 |
|  | 85 | PL9643 | 24 | 1.6 | -6.4, 9.5 | 3.930 |  |  |  |  |
| Itching | 15 | Placebo | 28 | -0.8 | -9.3, 7.8 | 4.250 | 0.8694 | 0.8792 | 0 | 6.58 |
|  | 15 | PL9643 | 24 | -1.7 | -10.9, 7.5 | 4.590 |  |  |  |  |
|  | 85 | Placebo | 26 | -3.9 | -11.6, 3.8 | 3.830 | 0.3861 | 0.4688 | -3.0 | 5.59 |
|  | 85 | PL9643 | 24 | -8.0 | -16.0, 0.0 | 3.990 |  |  |  |  |
| Foreign Body Sensation | 15 | Placebo | 28 | -2.1 | -9.6, 5.4 | 3.720 | 0.5901 | 0.3602 | -5.1 | 5.53 |
|  | 15 | PL9643 | 24 | -7.2 | -15.3, 0.9 | 4.030 |  |  |  |  |
|  | 85 | Placebo | 26 | -4.7 | -12.4, 3.0 | 3.810 | 0.9383 | 0.6546 | 2.5 | 5.58 |
|  | 85 | PL9643 | 24 | -2.2 | -10.2, 5.8 | 5.580 |  |  |  |  |
| Eye Discomfort | 15 | Placebo | 28 | 0.1 | -6.1, 6.4 | 3.110 | 0.1686 | 0.1134 | -7.4 | 4.59 |
|  | 15 | PL9643 | 24 | -7.3 | -14.0, -0.5 | 3.360 |  |  |  |  |
|  | 85 | Placebo | 26 | -8.4 | -15.8, -0.9 | 3.710 | 0.9307 | 0.8824 | 0.8 | 5.37 |
|  | 85 | PL9643 | 24 | -7.6 | -15.3, 0.2 | 3.860 |  |  |  |  |
| Eye Dryness | 15 | Placebo | 28 | -4.4 | -12.2, 3.5 | 3.920 | 0.3580 | 0.4421 | -4.5 | 5.77 |
|  | 15 | PL9643 | 24 | -8.8 | -17.3, -0.3 | 4.230 |  |  |  |  |
|  | 85 | Placebo | 26 | -6.5 | -14.5, 1.4 | 3.940 | 0.6569 | 0.7321 | -2.0 | 5.69 |
|  | 85 | PL9643 | 24 | -8.5 | -16.8, -0.2 | 4.110 |  |  |  |  |
| Photophobia | 15 | Placebo | 28 | -1.8 | -6.9, 3.3 | 2.540 | 0.9781 | 0.9574 | -0.2 | 3.74 |
|  | 15 | PL9643 | 24 | -2.0 | -7.5, 3.5 | 2.740 |  |  |  |  |
|  | 85 | Placebo | 26 | -1.7 | -9.5, 6.0 | 3.830 | 0.3758 | 0.6607 | -2.4 | 5.54 |
|  | 85 | PL9643 | 24 | -4.2 | -12.2, 3.8 | 3.990 |  |  |  |  |
| Pain | 15 | Placebo | 28 | -3.2 | -7.4, 1.0 | 2.070 | 0.6217 | 0.5816 | 1.7 | 3.05 |
|  | 15 | PL9643 | 24 | -1.5 | -6.0, 3.0 | 2.240 |  |  |  |  |
|  | 85 | Placebo | 26 | -1.3 | -8.1, 5.4 | 3.350 | 0.5819 | 0.3310 | -4.7 | 4.83 |
|  | 85 | PL9643 | 24 | -6.1 | -13.1, 0.9 | 3.480 |  |  |  |  |

ANCOVA, analysis of covariance; CAE, controlled adverse environment; LS, least squares; SE, standard error, WRS, Wilcoxon rank sum test.
